# Spatiotemporal regulation of liver development by the Wnt/β-catenin pathway

**DOI:** 10.1038/s41598-018-20888-y

**Published:** 2018-02-09

**Authors:** Zoë D. Burke, Karen R. Reed, Sheng-Wen Yeh, Valerie Meniel, Owen J. Sansom, Alan R. Clarke, David Tosh

**Affiliations:** 1 Centre for Regenerative Medicine, Department of Biology & Biochemistry, University of Bath, Claverton Down, Bath, BA2 7AY UK; 2 European Cancer Stem Cell Research Institute, Hadyn Ellis Building, Cardiff University, Cardiff, CF24 4HQ UK; 3 The Beatson Institute, Garscube Estate, Glasgow, G61 18D UK

## Abstract

While the Wnt/β-catenin pathway plays a critical role in the maintenance of the zonation of ammonia metabolizing enzymes in the adult liver, the mechanisms responsible for inducing zonation in the embryo are not well understood. Herein we address the spatiotemporal role of the Wnt/β-catenin pathway in the development of zonation in embryonic mouse liver by conditional deletion of *Apc* and *β-catenin* at different stages of mouse liver development. In normal development, the ammonia metabolising enzymes carbamoylphosphate synthetase I (CPSI) and Glutamine synthetase (GS) begin to be expressed in separate hepatoblasts from E13.5 and E15.5 respectively and gradually increase in number thereafter. Restriction of GS expression occurs at E18 and becomes increasingly limited to the terminal perivenous hepatocytes postnatally. Expression of nuclear β-catenin coincides with the restriction of GS expression to the terminal perivenous hepatocytes. Conditional loss of *Apc* resulted in the expression of nuclear β-catenin throughout the developing liver and increased number of cells expressing GS. Conversely, conditional loss of *β-catenin* resulted in loss of GS expression. These data suggest that the Wnt pathway is critical to the development of zonation as well as maintaining the zonation in the adult liver.

## Introduction

The adult liver exhibits a remarkable phenomenon known as metabolic zonation^1,2^. This refers to the heterogeneous distribution of key rate-limiting enzymes and metabolic pathways across the liver lobule or acinus^3^. The most striking example of zonation is observed in the enzymes and pathways of ammonia detoxification^4^. The urea cycle and the enzyme glutamine synthetase (GS) are two systems responsible for the removal of ammonia in the liver. Carbamoylphosphate synthetase I (CPSI) is the rate-limiting enzyme in the urea cycle and is expressed in the periportal, intermediate and first few layers of the perivenous zone. GS is expressed in a complementary fashion to CPSI and catalyses the ATP-dependent formation of glutamine from glutamate and ammonia. In the developing liver the onset and pattern of expression of CPSI and GS has been documented^5–7^. Expression of CPSI and GS is first detected in a small number of cells at embryonic day 13 (E13) and embryonic day 15 (E15) respectively, thereafter expression gradually increases throughout development with a decline in CPSI accumulation in the hepatocytes around the central vein occurring at E17.5^5^. In contrast, Shiojiri *et al*. documented that CPSI is first detected at E15 while GS is first expressed in the perivenous regions of the postnatal liver three days after birth^6,7^. This discrepancy has been attributed to the fixation conditions used during tissue preparation^5^. Both studies showed that the strict compartmentalization of these two enzymes observed in adult liver occurs postnatally.

The Wnt/β-catenin pathway has been proposed to play a key role in the maintenance of metabolic zonation in the adult liver^8,9^. Deletion of the Wnt effector *Apc*, which leads to ligand independent activation of β-catenin, or inhibition of Wnt signalling by virally-mediated hepatic overexpression of the inhibitor DKK1 disrupts the normal zonation of ammonia metabolizing enzymes^8^. Loss of *Apc* results in a reduction of CPSI and an expansion of GS expression. Deletion of *c-Myc* does not rescue the liver phenotype when *Apc* is deleted suggesting that β-catenin regulates zonation through a c-Myc-independent mechanism^9^. The sensitivity of hepatocytes to a threshold of Wnt/β-catenin signaling in the regulation of zonation has also been demonstrated in *Apc*^*min*^ mice in which expression of full length APC is partially attenuated^10^.

The Wnt/β-catenin pathway is also important in liver development. Hepatoblast-specific inactivation of *Apc*, through expression of Cre-recombinase under the control of a chimeric alpha-fetoprotein-albumin enhancer/promoter, results in the accumulation of β-catenin in the liver from E11.5 and subsequent embryonic lethality between E16.5 and E18.5^11^. *Apc* loss also resulted in liver hypoplasia and failure of hepatoblasts to differentiate towards the hepatocyte lineage. *Apc-*deficient liver also exhibited an increase in bile duct morphogenesis indicating a preference towards biliary differentiation in the presence of activated β-catenin^11^. Deletion of β*-catenin* in hepatoblasts resulted in significant underdevelopment of the liver from E12 with lethality occurring at around E17^12^. *β-catenin*-deficient embryonic liver lack hepatocyte and bile duct compartments. Collectively, these observations suggest a critical role for β-catenin in the survival, maturation and differentiation of hepatoblasts.

The early embryonic lethality observed in these studies has precluded a detailed analysis of the effects of disrupting the Wnt/β-catenin pathway on the expression of ammonia metabolizing enzymes in the developing mouse liver^11,12^. A role for the Wnt/β-catenin pathway in hepatic zonation of ammonia metabolizing enzymes has previously been established in adult liver but the question remains as to whether this pathway is also involved in establishing hepatic zonation during embryogenesis^8,9,13^. Here, we use inducible conditional deletion of *Apc* and *β-catenin* to dissect the role of Wnt/β-catenin signaling in the development of metabolic zonation of GS and CPSI.

## Results

### Expression of CPSI, GS and β-catenin in normal liver development

**Figure 1:**
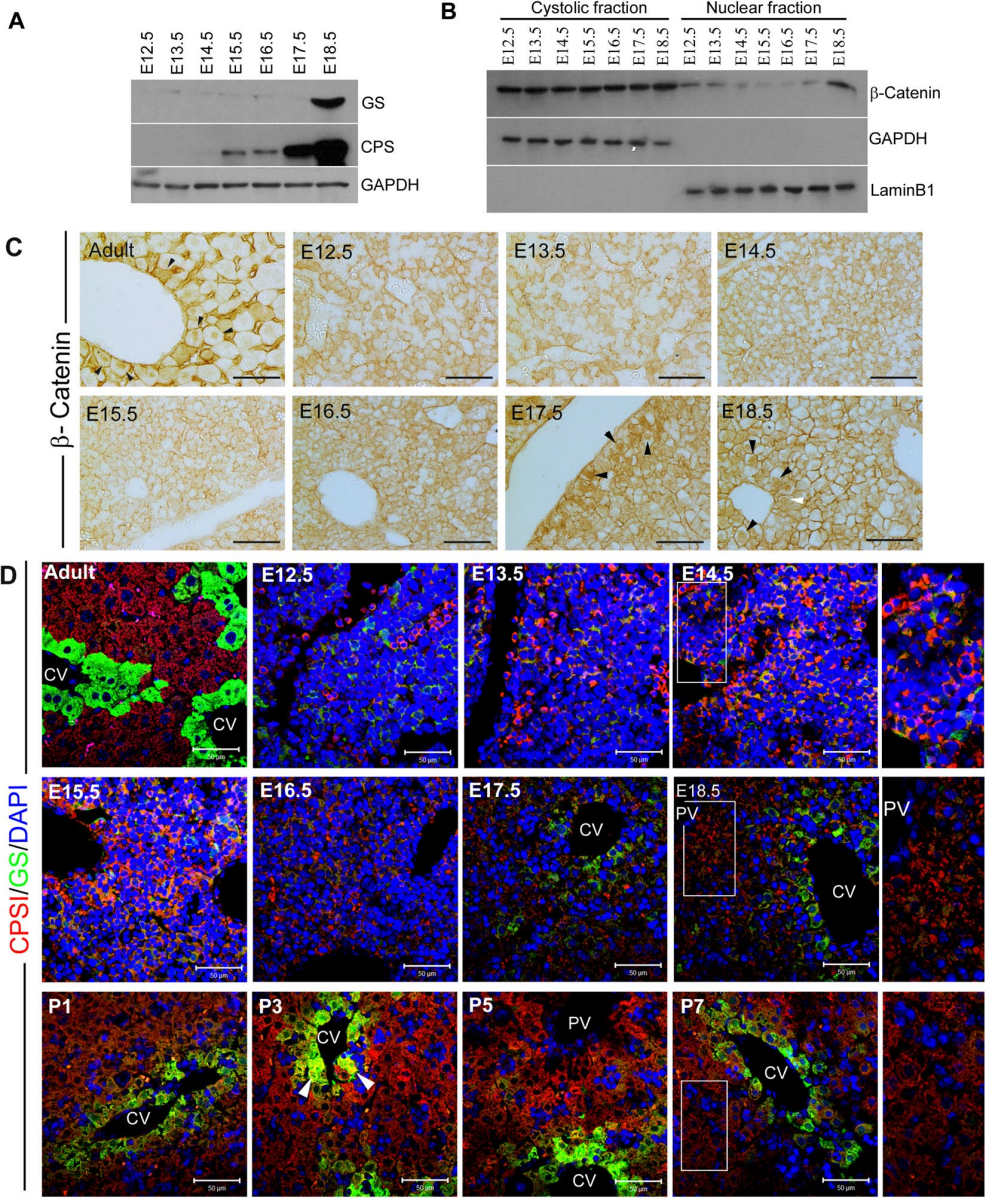
Timecourse of CPSI and GS protein expression levels and immunohistochemistry in developing mouse liver. (**A**) Western blot analysis to detect CPSI and GS protein in embryonic liver using GAPDH as a loading control. (**B**) Western blot analysis of cytoplasmic and nuclear protein fractions isolated from embryonic liver to show activation status of β-catenin using GAPDH and LaminB1 as loading controls. (**C**) Immunohistochemical staining for β-catenin in embryonic liver sections taken from the gestational stage indicated. The magnification is x400, scale bars −100 µm. (**D**) Immunofluorescent co-staining for CPSI (red) and GS (green) with DAPI counterstain (blue) in adult, embryonic and postnatal liver sections taken from the gestational/post gestational stage indicated. The magnification is x200 (inset is x400), scale bars −50 µm.

Previous studies have described conflicting reports on the appearance of CPSI and GS in the developing liver. Notenboom *et al*. described the onset of CPSI and GS expression at E13 and E15 with a gradual increase as development proceeds, followed by a decline in CPSI protein in hepatocytes surrounding the central vein prior to birth^5^. However, Shiojiri *et al*. observed that the onset of CPSI expression occurred at E15 while GS expression appeared postnatally^6^. The discrepancy in GS staining has been attributed to differences in tissue preparation while the observation that CPSI accumulation exhibits intralobular heterogeneity may account for differences observed in CPSI staining^5^. We first determined the expression pattern of CPSI and GS in relation to that of β-catenin at different stages of liver development (from E12.5 up to E18.5) in wild-type mice using immunostaining and western blotting (Fig. 1). As the specific antiserum used in the detection of CPSI and GS may also account for some differences we used a purified monoclonal antibody for the detection of GS and CPSI antibodies from two different sources. In line with the observations of previous reports, CPSI protein was found to be more highly expressed at E18.5 compared to earlier time points by Western blotting (Fig. 1A). Weak expression of GS protein was detected from E13.5 with higher levels detected only at E18.5 (Fig. 1A).

Given that β-catenin regulates GS expression in the adult liver we determined the pattern of total versus nuclear β-catenin to establish whether or not a change in activation status of the Wnt pathway coincided with the onset of GS expression during development. Although we found evidence of nuclear β-catenin throughout liver development (Fig. 1B), the highest levels were observed at E18.5 correlating with the peak of GS expression (Fig. 1A,B).

To ascertain when the normal embryonic expression pattern was established, we determined the localisation of CPSI, GS and β-catenin in the developing mouse liver. We found that many, but not all cells, exhibited strong positive staining for CPSI at E12.5. The number of positively-stained cells gradually increased, albeit showing a decrease in staining intensity, with all hepatoblasts expressing CPSI by E18.5 (Fig. 1D). We did not observe a well-defined decline in CPSI staining in the cells closely associated with the central vein. Staining for CPSI intensified between day 1 and day 5 postnatally with some hepatocytes adjacent to the central vein co-expressing GS (Fig. 1D; P3 white arrow heads), supporting the previously reported observation that zonation of CPSI is established postnatally^5^. We detected weak, diffuse and random staining for GS between E12.5–E16.5. Later, from E17.5 onwards, staining for GS intensified and became restricted to cells localised to discreet areas around the central vein. Cells surrounding the central vein also started to exhibit nuclear accumulation of β-catenin at this stage (Fig. 1C). While GS expression at E18.5 is not as severely restricted as in the adult liver, our data suggest that zonation of GS is established just prior to birth and that β-catenin may play a role in establishing the pattern of GS expression during development.

### Deletion of Apc and β-catenin disrupts the zonation of GS at E18.5

Previous studies of early β*-catenin* or *Apc* knockout in hepatoblasts resulted in mid to late gestational lethality^11,12^. The fact that lethality occurred between E16.5 and E18.5 made it difficult to determine the direct effect of aberrant activation of the Wnt/β-catenin pathway on development of metabolic zonation. To address this problem, we used a conditional approach for deletion of *Apc* and *β-catenin* by AhCre-mediated recombination. In the *AhCre* transgenic line, Cre expression is under the control of the Cyp1A1 promoter element that is transcriptionally up-regulated in response to lipophilic xenobiotics such as β-naphthoflavone (β-NF). The inducible *Ahcre* model provides an efficient approach for controlling Cre-mediated gene deletions in the hepatoblasts of the mouse liver in a time-specific manner. *Ahcre* mice were intercrossed with mice carrying Apc Apc580S (*Apc*^*fl*/*fl*^) or β-catenin (*β-Cat*^*fl*/*fl*^) loxP flanked alleles^14–16^. Pregnant females were identified and injected intraperitoneally with a single dose of β-NF at E13.5, E14.5 or E16.5 and embryonic tissue isolated either 2 or 4 days following recombination. We did not attempt inducing recombination at E13.5 and analysing at E18.5. Adult *Ahcre* + *Apcfl*/*fl* mice rapidly deteriorate within 4 days following APC deletion thus for consistency the latest we analysed genotypes was 4 days post-recombination.

**Figure 2:**
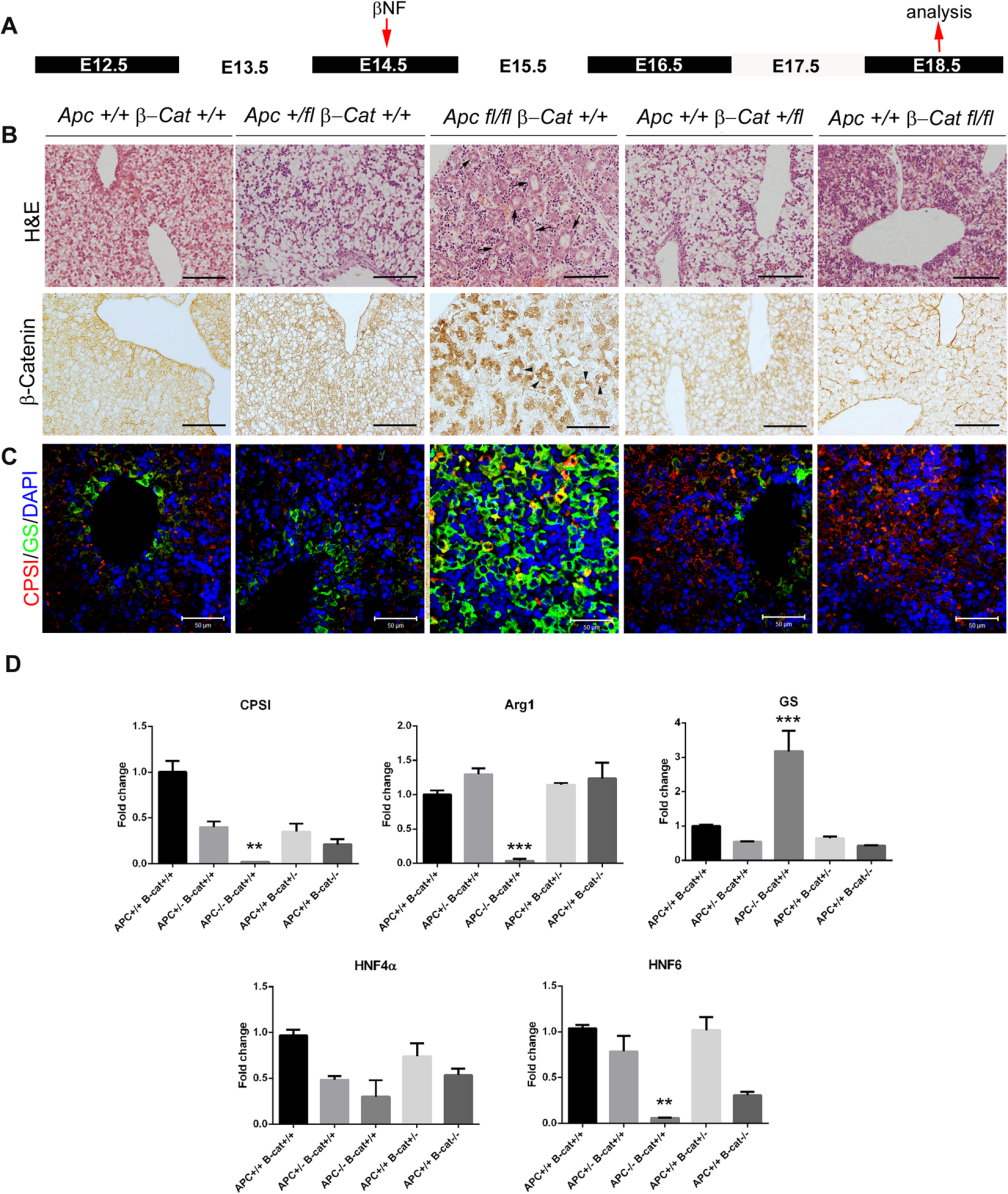
Immunohistochemical and qRT-PCR analysis of E18.5 embryonic liver following recombination at E14.5. (**A**) Schematic illustration of treatment regime showing time point of β-naphthoflavone injection (E14.5) and time point of tissue isolation for analysis (E18.5). (**B**) Haematoxylin & Eosin staining and immunohistochemical staining for β-catenin in embryonic liver sections taken from *AhCre*^+^*Apc*^+/+^*β-Cat*^+*l*+^*, AhCre*^+^*Apc*^+/*fl*^*β-Ca*t^+l+^*, AhCre*^+^*Apc*^*fl*/*fl*^*β-Cat*^+1+^*, AhCre*^+^*Apc*^+/+^*β-Cat*^+*lfl*^ and *AhCre*^+^*Apc*^+/+^*β-Cat*^*fllfl*^ recombinants. (**C**) Immunofluorescent co-staining for CPSI (red) and GS (green) with DAPI counterstain (blue) in embryonic liver sections taken from the recombinants indicated. The magnification is x200, scale bars −50 µm. (**D**) qRT-PCR analysis of cDNA isolated from E18.5 embryonic liver following deletion of *Apc* and *β-catenin* at E14.5. Statistical changes and *P* values are indicated (***p < 0.0005; **p < 0.005).

**Figure 3:**
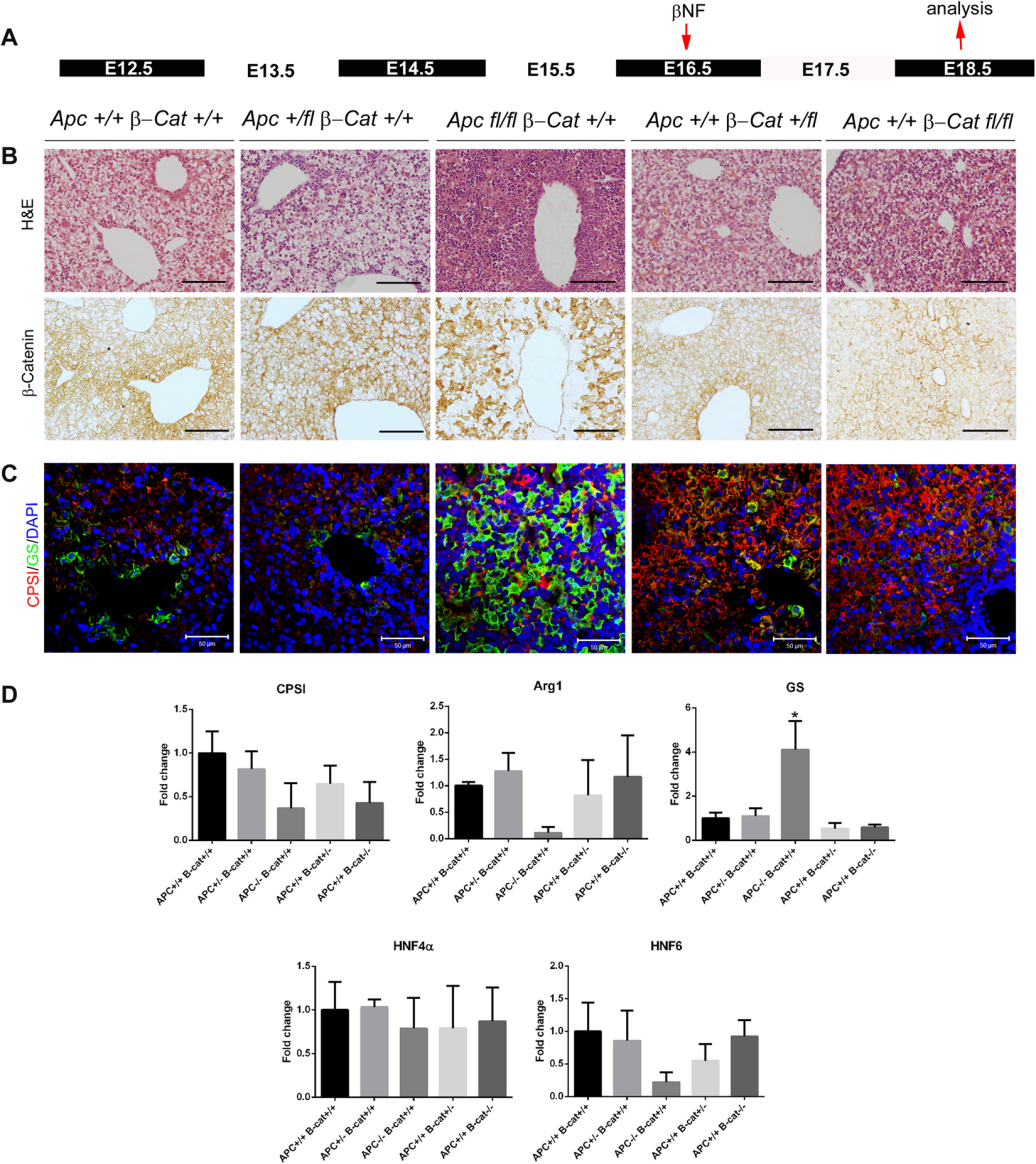
Immunohistochemical and qRT-PCR analysis of E18.5 embryonic liver following recombination at E16.5. (**A**) Schematic illustration of treatment regime showing time point of β-naphthoflavone injection (E16.5) and time point of tissue isolation for analysis (E18.5). (**B**) Haematoxylin & Eosin staining and immunohistochemical staining for β-catenin in embryonic liver sections taken from *AhCre*^+^*Apc*^+/+^*β-Cat*^+*l*+^*, AhCre*^+^*Apc*^+/*fl*^*β-Cat*^+*l*+^*, AhCre*^+^*Apc*^*fl*/*fl*^*β-Cat*^+*l*+^*, AhCre*^+^*Apc*^+/+^*β-Cat*^+*lfl*^ and *AhCre*^+^*Apc*^+/+^*β-Cat*^*fllfl*^ recombinants. (**C**) Immunofluorescent co-staining for CPSI (red) and GS (green) with DAPI counterstain (blue) in embryonic liver sections taken from the recombinants indicated. The magnification is x200, scale bars −50 µm. (**D**) qRT-PCR analysis of cDNA isolated from E18.5 embryonic liver following deletion of *Apc* and *β-catenin* at E16.5. Statistical changes and *P* values are indicated (*p < 0.05).

To determine whether β-catenin is responsible for establishing zonation, β-NF was first injected into pregnant female mice at either E14.5 or E16.5 (Figs 2A and 3A respectively) and embryos were then isolated at E18.5. To confirm the efficient loss of *Apc* and β*-catenin*, immunohistochemistry for β-catenin was performed on different recombinants: *AhCre*^+^*Apc*^+/+^*β-Cat*^+*l*+^, *AhCre*^+^*Apc*^+/*fl*^*β-Cat*^+*l*+^, *AhCre*^+^*Apc*^*fl*/*fl*^*β-Cat*^+*l*+^, *AhCre*^+^*Apc*^+/+^*β-Cat*^+*lfl*^ and *AhCre*^+^*Apc*^+/+^*β-Cat*^*fllfl*^ (Figs 2B and 3B – for gross morphology similar sections stained with Haematoxylin and Eosin are also shown). Nuclear accumulation of β-catenin was observed throughout the liver of *AhCre*^+^*Apc*^*fl*/*fl*^*β-Cat*^+*l*+^ embryos, this was accompanied by an increase in the number of cells staining robustly for GS (Fig. 2C) and a 3.9-fold increase in GS mRNA (Fig. 2D). The normal pattern of CPSI expression is also disrupted with the majority of hepatocytes showing little or no staining, although a few cells, possibly due to lack of recombination, continue to express CPSI staining very intensely and co-stain with GS (Fig. 2C). Quantitative reverse transcription polymerase chain reaction (qRT-PCR) analysis confirmed this observation. A second periportal gene, *Arginase 1* (*Arg1*), and the liver enriched transcription factors *Hnf4α* and *Hnf6* (Fig. 2D) also showed reduced gene expression by qRT-PCR (23-, 2.5- and 15.2-fold respectively). Intriguingly, histological analysis of haematoxylin and eosin-stained liver sections from *AhCre*^+^*Apc*^*fl*/*fl*^*β-Cat*^+*l*+^ liver revealed the presence of duct-like structures (Fig. 2B; arrows). These structures exhibit intense staining for nuclear β-catenin (Fig. 2B; arrowheads) and GS. Deletion of *β-catenin* by recombination at E14.5 resulted in loss of zonation of GS (Fig. 2C), a 1.9-fold reduction in *GS* mRNA levels (Fig. 2D) and increased staining for CPSI (Fig. 2C). However, levels of CPSI mRNA were reduced 3.7-fold in *AhCre*^+^*Apc*^+/+^*β-Cat*^*fllfl*^ and accompanying these changes a reduction in expression of *Hnf6* (2.9-fold) and *Hnf4α* (1.4-fold) was detected (Fig. 2D; these changes were not significant).

Deletion of *Apc* by recombination at E16.5 resulted in nuclear accumulation of β-catenin, expansion of GS expression throughout the liver of E18.5 *AhCre*^+^*Apc*^*fl*/*fl*^*β-Cat*^+*l*+^ embryos (Fig. 3B,C) and a 4.1-fold increase in GS mRNA levels (Fig. 3D). In contrast, the pattern of CPSI expression showed little change except that individual cells strongly expressing CPSI were scattered across the liver (Fig. 3C). Interestingly, *Arg1* and *Hnf6* showed a reduction in expression, in line with changes observed following *Apc* deletion at E14.5, while *Hnf4α* showed only a slight (1.3-fold) decrease in gene expression. Immunohistochemical analysis of *AhCre*^+^*Apc*^+/+^*β-Cat*^*fllfl*^ liver following recombination at E16.5 revealed the loss of GS zonation. The pattern of CPSI staining remained unchanged but the intensity of staining was increased throughout the liver. However, qPCR showed a reduction in mRNA levels of 1.7-fold and 2.3-fold for *GS* and *CPSI* respectively (Fig. 3D). Heterozygous deletion of *Apc* (*AhCre*^+^*Apc*^+/*fl*^*β-Cat*^+/+^), following recombination at E14.5 and E16.5, resulted in a similar pattern of staining for GS and CPSI to that observed in the wild type liver and nuclear staining for β*-*catenin was marginally more widespread. Conversely fewer cells exhibited nuclear β*-*catenin staining in *AhCre*^+^*Apc*^+/+^*β-Cat*^+*lfl*^ liver sections accompanied by less robust staining for GS following recombination at both gestational time points. No significant differences were observed by qRT-PCR in *GS* and *CPSI* levels. Thus overall these results show that dose-dependent disruption of Wnt signalling either by ablation or activation of β-catenin affects both the pattern and intensity of CPSI expression and results in disruption of GS.

### Deletion of Apc at E13 induces ectopic expression of GS

**Figure 4:**
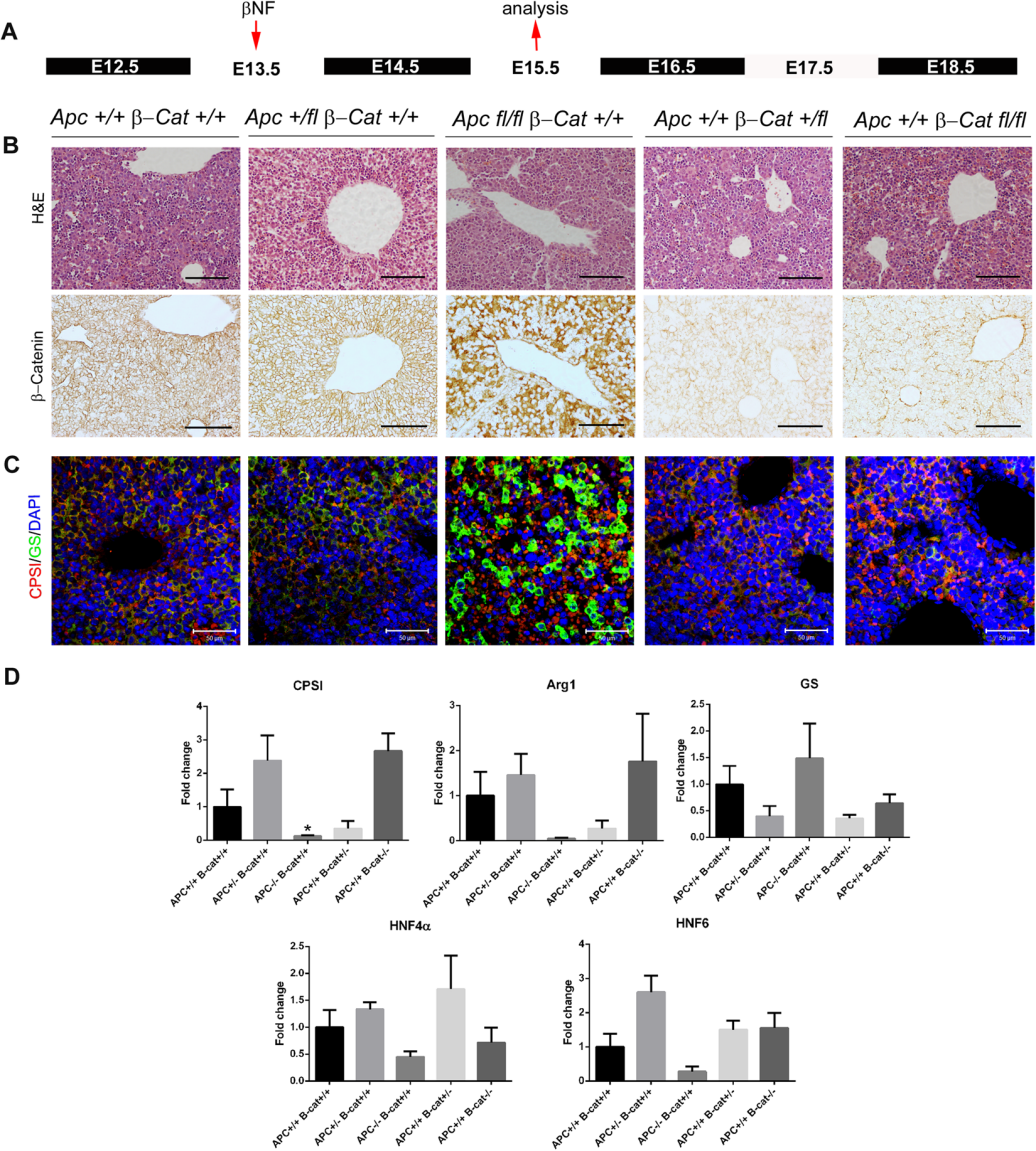
Immunohistochemical and qRT-PCR analysis of E15.5 embryonic liver following recombination at E13.5. (**A**) Schematic illustration of treatment regime showing time point of β-naphthoflavone injection (E13.5) and time point of tissue isolation for analysis (E15.5). (**B**) Haematoxylin & Eosin staining and immunohistochemical staining for β-catenin in embryonic liver sections taken from *AhCre*^+^*Apc*^+/+^*β-Cat*^+*l*+^*, AhCre*^+^*Apc*^+/*fl*^*β-Cat*^+*l*+^*, AhCre*^+^*Apc*^*fl*/*fl*^*β-Cat*^+*l*+^*, AhCre*^+^*Apc*^+/+^*β-Cat*^+*lfl*^ and *AhCre*^+^*Apc*^+/+^*β-Cat*^*fllfl*^ recombinants. (**C**) Immunofluorescent co-staining for CPSI (red) and GS (green) with DAPI counterstain (blue) in embryonic liver sections taken from the recombinants indicated. The magnification is x200, scale bars −50 µm. (**D**) qRT-PCR analysis of cDNA isolated from E15.5 embryonic liver following deletion of *Apc* and *β-catenin* at E13.5. Statistical changes and *P* values are indicated (*p < 0.05).

To investigate the possibility that activation of β-catenin, at earlier stages in development, might induce premature expression of GS, we induced recombination at E13.5 (Fig. 4) and isolated the liver prior to the onset of GS zonation at E15.5. In wild-type livers at E15.5, we did not observe clear nuclear staining for β-catenin and only diffuse, weak random staining for GS. In *AhCre*^+^*Apc*^*fl*/*fl*^*β-Cat*^+*l*+^ embryos following recombination at E13.5 we saw robust nuclear accumulation of β-catenin (Fig. 4B) accompanied by strong staining for GS throughout the liver (Fig. 4C) and a concomitant 1.5-fold increase in *GS* mRNA levels (Fig. 4D). While the pattern of CPSI staining remained largely unaltered in the *AhCre*^+^*Apc*^*fl*/*fl*^*β-Cat*^+*l*+^ liver, an 8.2-fold decrease in *CPSI* mRNA levels was observed (Fig. 4D). *Arg1*, *Hnf4α* and *Hnf6* all exhibited a reduction in expression (21-fold, 2.2-fold and 3.7-fold respectively (Fig. 4D). *AhCre*^+^*Apc*^+/*fl*^*β-Cat*^+*l*+^ liver sections exhibited similar staining for β*-*catenin as the wild type liver at E15.5. No significant changes in GS and CPSI mRNA levels were observed. Homozygous and heterozygous deletion of *β-catenin* at E13.5 reduced β-catenin expression but did not alter GS or CPSI immunostaining in E15.5 liver (Fig. 4B,C). By qRT-PCR analysis, *GS* mRNA levels were unaltered while *CPSI* and *Arg1* expression was increased (2.6- and 1.8-fold respectively) and *Hnf4α* expression was reduced (1.4-fold) (Fig. 4D). This data suggests that activation of β-catenin prior to the normal onset of expression is sufficient for premature induction of GS expression in the developing liver.

## Discussion

The zonal regulation of ammonia metabolising enzymes in the adult liver is controlled by the Wnt/β-catenin pathway^8,9,13^. However, the mechanism involved in regulating the zonal expression of these enzymes in the developing liver remain unclear. Previously, Notenboom *et al*. established that the expression of CPSI begins at around E13.5 and becomes expressed throughout the liver by E18.5. Our data is consistent with these observations. Restriction of expression of CPSI from the terminal perivenous hepatocytes, as seen in adult liver, is reported to occur postnatally at weaning^17^. In contrast, the onset of expression of GS is less certain with its first appearance being reported from E15.5 up to 3 days postnatally^5,6^. We observed the robust and ordered induction of GS expression at E17.5-E18.5. Expression of GS and nuclear β-catenin were observed within the same cells in discrete areas of the liver (associated with vascular structures). For the first time, our data provides evidence that the zonation of GS during development is regulated through the Wnt/β-catenin pathway. Previously, *Apc* deletion in mouse embryos at E11.5 caused severe liver hypoplasia, a reduction in expression of hepatoblast/hepatocyte markers (*α-fetoprotein*, *albumin*, *transthyretin* and *CPSI*) and embryonic lethality between E16.5–E18.5^11^. The authors also showed a partial commitment toward the biliary lineage, based on the presence of duct-like structures overexpressing the biliary marker integrin β4^11^.

To determine in more detail the dependence of *GS* expression on β-catenin activation, we used the previously reported *AhCre Apc* model. Here, Cre transgene expression is driven by the inducible *Cyp1A1* promoter^9^. All of the embryos (littermates) came from mothers that were treated with β-napthoflavone. We did not use animals that were either untreated or AhCre^−^. The observed changes in zonation therefore correlate with genotype (reduced gene expression) and not with the treatment. We cannot exclude the possibility that the changes are due to hypomorphic effects of the floxed alleles. Disruption of *Apc* at each of the time-points examined, resulted in nuclear β-catenin in the majority of cells throughout the embryonic liver demonstrating efficient recombination and activation of the Wnt signalling pathway. A concomitant increase in GS staining and mRNA levels was revealed by immunohistochemical and qPCR analysis respectively for all time points examined. Following recombination at E14.5, we observed a complementary loss of CPSI staining and gene expression in E18.5 livers suggesting a disposition for hepatoblasts to become perivenous-like at the expense of the periportal phenotype. We also examined the effect of forced β-catenin activation at E13.5 and found that GS expression was induced in the E15.5 liver (i.e. earlier than normal) while *CPSI* mRNA levels were reduced. Interestingly, recombination at E16.5 did not alter CPSI expression (either at the protein or mRNA level) indicating differential sensitivity of the periportal phenotype to β-catenin activation in a temporal manner. This difference may be due, in part, to the expression of *Hnf4α*. It has been proposed that Hnf4α plays a crucial role in regulating zonation in adult mouse liver^18,19^. In one of these studies, Hnf4α directly binds to the upstream enhancer of *GS* suggesting that suppression of GS expression in periportal hepatocytes is mediated through Hnf4α-dependent recruitment of repressor complexes^18^. In a complementary study, expression of *GS* was repressed by the binding of Hnf4α to its own consensus sequence within the promoter^19^. Hnf4α-mediated repression is inhibited upon β-catenin activation through the displacement of Hnf4α by the Wnt signalling mediator LEF1. However, activation of the Wnt pathway and binding of LEF1 results in the displacement of Hnf4α and a consequent decline in expression^19^. These studies reveal a dependence of zonation on the convergence of Hnf4α on Wnt signalling pathway. In the context of our study, the relatively unaltered levels of *Hnf4α* mRNA following activation of β-catenin at E16.5, may be responsible for the observed lack of change in *CPSI* expression. In addition, the persistent staining for CPSI in the *Apc* null liver following recombination at E16.5 may be due to stability of the enzyme.

Inactivation of the Wnt signalling pathway in the developing mouse liver has been accomplished through conditional deletion of β-catenin^12^. *Foxa3 Cre β-catenin*^*fl*/*fl*^ embryos exhibit a reduction in liver size and embryonic lethality occurring at E17.5. Crucially, we have extended these studies to determine the effect of deletion of β-catenin on expression and zonation of GS in a conditional and temporally controlled manner. *β-catenin* deletion at E14.5 and E16.5 caused almost complete loss of GS staining and a reduction in gene expression in the E18.5 liver, demonstrating a requirement for β-catenin in induction and zonation of GS during development. While CPSI staining in the E15.5 liver remains unchanged following recombination at E13.5, mRNA levels were elevated. Conversely, inactivation of Wnt signalling at E14.5 and E16.5 caused decreased *CPSI* gene expression at E18.5. These observations imply that maturation of hepatoblasts toward a periportal phenotype is influenced by the status of β-catenin activation between E14.5-E18.5. This was unexpected given that previous work supported the idea that the maintenance of the periportal phenotype in the adult requires inhibition of β-catenin^8,9^. During normal development, bi-potential hepatoblasts at E13.5 begin to differentiate to hepatocytes or biliary cells. The disruption of Wnt signalling either through stabilisation or inhibition of β-catenin prior to this stage has a profound effect on liver development by impairing normal hepatoblast fate decisions^8,12^. Stabilisation of β-catenin appears to drive hepatoblasts toward a biliary fate. In the present study, we noted the presence of duct-like structures in *AhCre Apc*^*fl*/*fl*^ embryos following recombination at E14.5. Ectopic staining and increased gene expression for the biliary progenitor marker Sox9 was observed following activation of β-catenin (Supplementary Figure 1), suggesting hepatoblasts were being pushed towards a biliary lineage.

Our data indicates that activated β-catenin is present throughout liver development albeit at very low levels during the early stages. Such temporal regulation is likely to play a role in biliary differentiation. However, the surge in β-catenin activation at E17.5-E18.5 marks the onset of hepatocyte differentiation toward a perivenous phenotype. A similar correlation between β-catenin activation and tissue maturation has been observed in intestinal development. Treatment of fetal intestinal tissue with Wnt3a upregulates the Wnt target gene Lgr5 and subsequent maturation towards the adult state occurs through cells exhibiting highest levels of Lgr5 expression^20^.

In the present study, we demonstrated an absolute dependence on β-catenin for the induction and patterning of GS expression in the developing liver. Moreover, dose-dependent and temporal regulation of β-catenin activation is likely to be critical as both activation and ablation *in vivo* of β-catenin results in disruption of normal GS expression. Furthermore our data, and that of a previous study^11^, have shown that misplaced activation of β-catenin during liver development can influence cell lineage decisions. Herein we propose that β-catenin signalling not only contributes to fate determination of the hepatoblast but also to the phenotype of the maturing hepatocyte^21,22^.

## Materials and Methods

### Mouse Lines

The loxP flanked alleles used were as follows *Apc*^15^, *β-catenin* and *AhCre*^14^. To induce recombination pregnant mice were given a single intraperitoneal injection of β-napthoflavone (β-NF) (80 mg/kg) at the gestational time points indicated (E13.5, E14.5 or E16.5). Tissue samples were harvested either 2 or 4 days after recombination. Mice were humanely killed by a schedule 1 procedure. Ethical approval was granted from Cardiff University’s Animal Welfare and Ethical Review Body (previously known as the ERP), and all animal procedures were conducted in accordance with UK Home Office regulations.

### Tissue Fixation, Embedding, and Processing

Embryonic liver tissue was fixed and processed as described previously^9^.

### Immunohistochemical and Immunofluorescent Analysis of Mouse Liver

Immunostaining for GS, CPSI, and β-catenin was carried out as described previously^9^ with the following changes;

High temperature antigen retrieval was performed in EDTA buffer for CPSI, GS, and β-catenin. Following antigen retrieval sections were immuno-histochemically labelled for β-catenin or immunofluorescently labelled for CPSI and GS.

For immunohistochemistry sections were blocked in 10% normal goat serum (NGS) and 1% bovine serum albumin (BSA) in PBS. Antibodies were diluted in 1% NGS/1%BSA in PBS: anti-mouse β-catenin (1:50; Transduction Laboratories, San Diego, CA). Antibody detection was carried out using HRP conjugated secondary antibodies (1:200; Vector labs) and DAB (Vector Labs). All immunohistochemical staining was imaged using an Olympus microscope and CellP Olympus software.

For immunofluorescent staining with CPSI and GS sections were blocked in 5% BSA/PBS. Antibodies were diluted in 1% BSA/PBS CPSI (1:200, a generous gift from Wouter Lamers), GS (1:500, Transduction Labs), anti-mouse Alexa 488 (1:1000, Molecular Probes, Invitrogen) and anti-rabbit Alexa 594 (1:500, Molecular Probes, Invitrogen). Fluorescent staining was imaged using a Zeiss LSM Meta confocal microscope and LSM software.

### Reverse Transcription and Polymerase Chain Reaction

RNA extraction, 1^st^ strand cDNA synthesis and reverse transcriptase polymerase chain reaction (RT-PCR) was performed as described previously^23^. Primer sequences used are listed in Table 1 below.

**Table 1:** Primer sequences used for reverse transcription and polymerase chain reaction.

	Forward Primer 5′-3′	Reverse Primer 5′-3′
β-Actin	TAGGCACCAGGGTGTGATGG	CATGGCTGGGGTGTTGAAGG
GS	TATTACTGCGGTGTGGGAGCA	TGAAGTTGGTATGGCAGCCT
HNF4α	CTCTTCTGATTATAAGCTGAGGAT	CCACAGGAAGGTGCAGATTGATCTG
CPSI	TGAGTGGGTCTGCCATGAAC	TCATCCCGCATAAGTGTGAA

### Fractionation of liver proteins and Western Blotting

Fractionation of liver proteins was performed as follows; liver tissue was lysed and homogenized in 500 μl of cytosolic extraction buffer (10 mM KCl, 20 mM HEPES, 1 mM EDTA, 10% Glycerol, pH7.9). The lysate was rotated at 4 °C for 30 min and further homogenized by 10 passes through a 27 G needle and syringe. The nuclear fraction was isolated by centrifugation at 3000 *G* for 5 min at 4 °C. The supernatant (containing the cytosolic fraction) was stored at −80 °C. The nuclear pellet was washed twice in 500 μl of cytosolic extraction buffer, dissolved in 50 μl nuclear extraction buffer (10 mM KCl, 20 mM HEPES, 400 mM NaCl, 1 mM EDTA, 20%Glycerol, pH 7.9) and rotated at 4 °C for 60 min. The lysate was centrifuged at 16300 G for 15 min at 4 °C and the supernatant (containing the nuclear fraction) was stored at −80 °C. Western blotting was carried out as previously described^23^. Antibodies used were obtained and diluted as follows: anti-mouse β-catenin (1:5000, Transduction Laboratories, San Diego, CA), anti-mouse GS (1:15000, Transduction Laboratories, San Diego, CA), anti-rabbit CPSI (1:15000, Abcam). Anti-mouse glyceraldehyde-3-phosphate dehydrogenase (GAPDH; 1:2000; Ambion), anti-goat-Lamin B1 (1:2000, Santa Cruz) and peroxidase labelled anti-rabbit or anti-mouse both at 1:2000 (Amersham Biosciences, Bucks, UK). The signal was detected with the ECL^TM^ Western blotting analysis system (Amersham) and developed on Hyperfilm^TM^ (Amersham). Autoradiographs were scanned on a Sharp MX-3570 printer/scanner and images manipulated using Photoshop Elements 10. Original uncropped autoradiographs are provided in Supplementary Figures 2 and 3.

### Data availability

All data generated or analysed during this study are included in this published article.

## Electronic supplementary material

Supplementary information

## References

[1] Gebhardt R (1992). Metabolic zonation of the liver: regulation and implications for liver function. Pharmacology & therapeutics.

[2] Kietzmann T (2017). Metabolic zonation of the liver: The oxygen gradient revisited. Redox biology.

[3] Tosh D, Alberti GM, Agius L (1988). Glucagon regulation of gluconeogenesis and ketogenesis in periportal and perivenous rat hepatocytes. Heterogeneity of hormone action and of the mitochondrial redox state. The Biochemical journal.

[4] Gebhardt R, Lindros K, Lamers WH, Moorman AF (1991). Hepatocellular heterogeneity in ammonia metabolism: demonstration of limited colocalization of carbamoylphosphate synthetase and glutamine synthetase. European journal of cell biology.

[5] Notenboom RG, Moorman AF, Lamers WH (1997). Developmental appearance of ammonia-metabolizing enzymes in prenatal murine liver. Microscopy research and technique.

[6] Shiojiri N (1995). Heterogeneous hepatocellular expression of glutamine synthetase in developing mouse liver and in testicular transplants of fetal liver. Laboratory investigation; a journal of technical methods and pathology.

[7] Shiojiri N, Wada J, Gebhardt R (1998). Heterogeneous carbamoylphosphate synthetase I expression in testicular transplants of fetal mouse liver. European journal of cell biology.

[8] Benhamouche S (2006). Apc tumor suppressor gene is the “zonation-keeper” of mouse liver. Developmental cell.

[9] Burke, Z. D. *et al*. Liver zonation occurs through a beta-catenin-dependent, c-Myc-independent mechanism. *Gastroenterology* **136**, 2316–2324 e2311–2313, 10.1053/j.gastro.2009.02.063 (2009). 10.1053/j.gastro.2009.02.063 19268669

[10] Buchert M (2010). Genetic dissection of differential signaling threshold requirements for the Wnt/beta-catenin pathway *in vivo*. PLoS genetics.

[11] Decaens T (2008). Stabilization of beta-catenin affects mouse embryonic liver growth and hepatoblast fate. Hepatology.

[12] Tan X (2008). Beta-catenin deletion in hepatoblasts disrupts hepatic morphogenesis and survival during mouse development. Hepatology.

[13] Planas-Paz L (2016). The RSPO-LGR4/5-ZNRF3/RNF43 module controls liver zonation and size. Nature cell biology.

[14] Ireland H (2004). Inducible Cre-mediated control of gene expression in the murine gastrointestinal tract: effect of loss of beta-catenin. Gastroenterology.

[15] Shibata H (1997). Rapid colorectal adenoma formation initiated by conditional targeting of the Apc gene. Science.

[16] Sansom OJ (2004). *Loss of Apc in vivo im*mediately perturbs Wnt signaling, differentiation, and migration. Genes & development.

[17] Gaasbeek Janzen JW, Moorman AF, Lamers WH, Charles R (1985). Development of the heterogeneous distribution of carbamoyl-phosphate synthetase (ammonia) in rat-liver parenchyma during postnatal development. The journal of histochemistry and cytochemistry: official journal of the Histochemistry Society.

[18] Stanulovic VS (2007). Hepatic HNF4alpha deficiency induces periportal expression of glutamine synthetase and other pericentral enzymes. Hepatology.

[19] Colletti M (2009). Convergence of Wnt signaling on the HNF4alpha-driven transcription in controlling liver zonation. Gastroenterology.

[20] Fordham RP (2013). Transplantation of expanded fetal intestinal progenitors contributes to colon regeneration after injury. Cell stem cell.

[21] Monga SP (2003). Beta-catenin antisense studies in embryonic liver cultures: role in proliferation, apoptosis, and lineage specification. Gastroenterology.

[22] Yang, J. *et al*. Beta-catenin signaling in murine liver zonation and regeneration: A Wnt-Wnt situation! *Hepatology*, 10.1002/hep.27082 (2014). 10.1002/hep.27082 PMC4139486 24700412

[23] Burke ZD, Shen CN, Ralphs KL, Tosh D (2006). Characterization of liver function in transdifferentiated hepatocytes. Journal of cellular physiology.

