# Author Correction: Spatiotemporal regulation of liver development by the Wnt/β-catenin pathway

**DOI:** 10.1038/s41598-020-68277-8

**Published:** 2020-07-02

**Authors:** Zoë D. Burke, Karen R. Reed, Sheng-Wen Yeh, Valerie Meniel, Owen J. Sansom, Alan R. Clarke, David Tosh

**Affiliations:** 1 Centre for Regenerative Medicine, Department of Biology and Biochemistry, University of Bath, Claverton Down, Bath, BA2 7AY UK; 2 European Cancer Stem Cell Research Institute, Hadyn Ellis Building, Cardiff University, Cardiff, CF24 4HQ UK; 3 The Beatson Institute, Garscube Estate, Glasgow, G61 18D UK

Correction to: *Scientific Reports* 10.1038/s41598-018-20888-y, published online 09 February 2018

This article contains an error in Figure 1B and the corresponding Supplementary Figure S3. A mistake in the labelling of the original autoradiograph resulted in the wrong image being included as the control blot in these Figures. A corrected Figure 1 and Supplementary Figure S3 appear below as Figure 1 and Figure 2 respectively.

**Figure 1:**
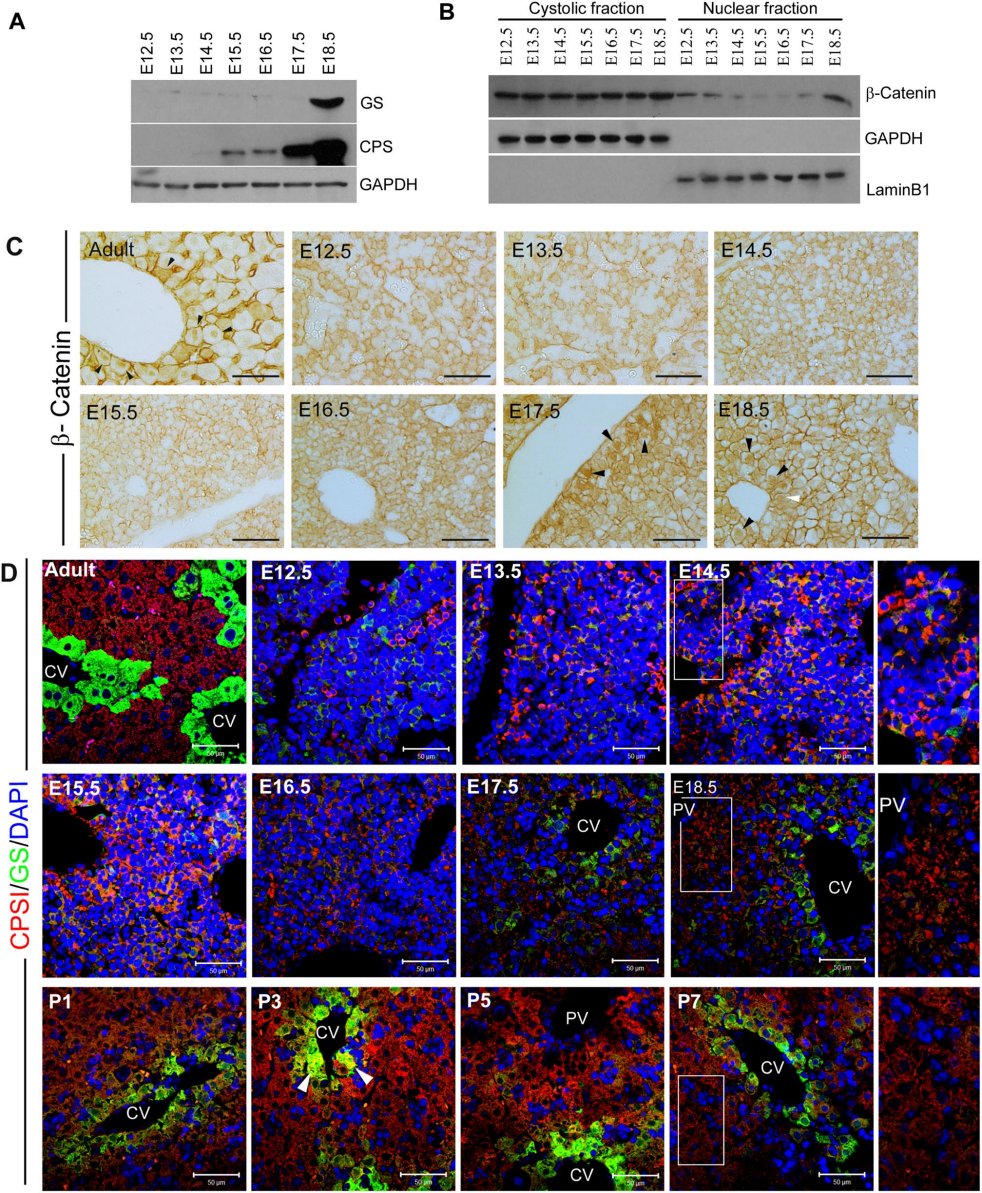
Timecourse of CPSI and GS protein expression levels and immunohistochemistry in developing mouse liver. (A) Western blot analysis to detect CPSI and GS protein in embryonic liver using GAPDH as a loading control. (B) Western blot analysis of cytoplasmic and nuclear protein fractions isolated from embryonic liver to show activation status of β-catenin using GAPDH and LaminB1 as loading controls. (C) Immunohistochemical staining for β-catenin in embryonic liver sections taken from the gestational stage indicated. The magnification is x400, scale bars −100 μm. (D) Immunofluorescent co-staining for CPSI (red) and GS (green) with DAPI counterstain (blue) in adult, embryonic and postnatal liver sections taken from the gestational/post gestational stage indicated. The magnification is ×200 (inset is ×400), scale bars −50 μm.

**Figure 2:**
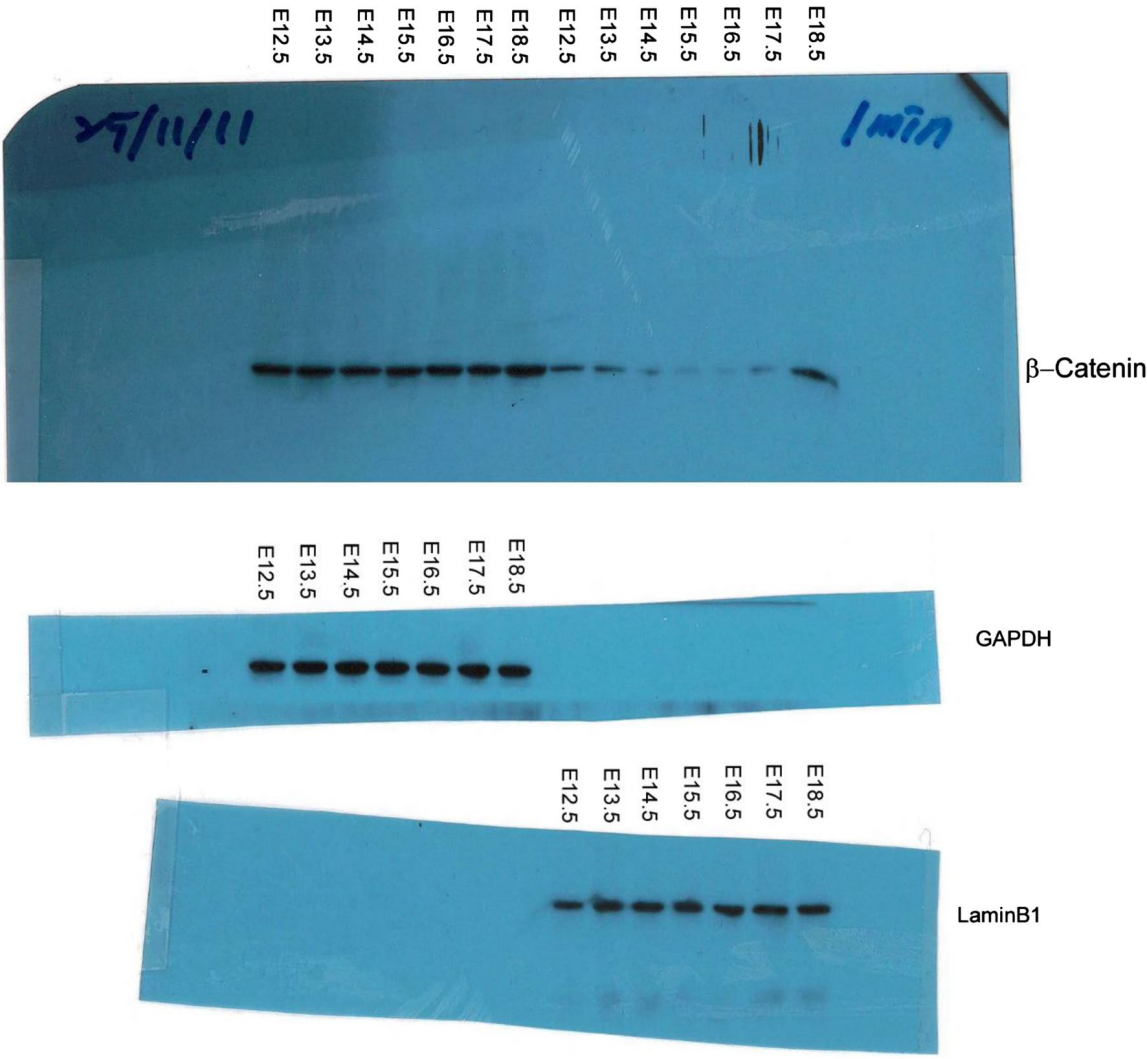
Scanned images of original autoradiographs showing Western Blot analysis for β-Catenin, GAPDH and LaminB1 in embryonic liver samples taken at the time points indicated (see Figure 1B).

