# Supplementary material for: Spatiotemporal regulation of liver development by the Wnt/β-catenin pathway
Source: Sci Rep. 2018 Feb 9;8:2735. doi: 10.1038/s41598-018-20888-y (PMC5807466; doi:10.1038/s41598-018-20888-y)
Supplement: Supplementary file 1 — Supplementary information [file 41598_2018_20888_MOESM1_ESM.pdf]

Spatiotemporal regulation of liver development by the Wnt/β-catenin pathway

Short title: beta-catenin regulates zonation development

Zoë D. Burke[1‡*], Karen R. Reed[2‡], Sheng-Wen Yeh[1], Valerie Meniel[2], Owen J. Sansom[3], Alan R. Clarke[2,] and David Tosh[1,*]

Supplementary Figure 1

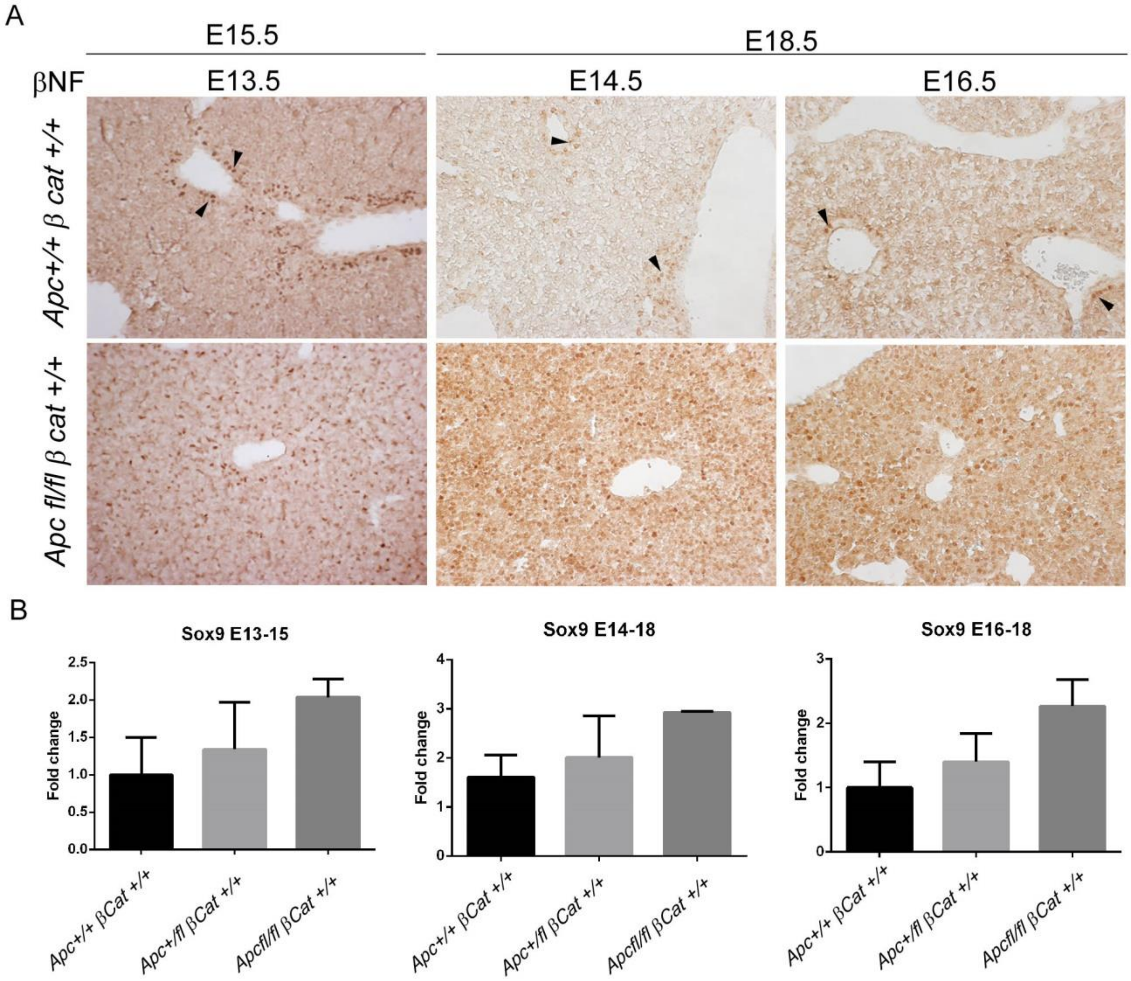

Supplementary Figure 1. Immunohistochemical and qRT-PCR analysis of Sox9 expression following deletion of *Apc.* (A) Immunohistochemical staining for Sox9 in *AhCre*$^{+}$*Apc*$^{+/+}$*β-Cat*$^{+/+}$ and *AhCre*$^{+}$*Apc*$^{fl/fl}$*β-Cat*$^{+/+}$ liver at E15.5 and E18.5 following induction of recombination at E13.5, E14.5 or E16.5. (B) qRT-PCR analysis of cDNA isolated from *AhCre*$^{+}$*Apc*$^{+/+}$*β-Cat*$^{+/+}$, *AhCre*$^{+}$*Apc*$^{+/fl}$*β-Cat*$^{+/+}$ and *AhCre*$^{+}$*Apc*$^{fl/fl}$*β-Cat*$^{+/+}$ recombinants. The magnification is x100. Immunostaining procedure was carried out with anti-rabbit Sox9 (1:100; Millipore) as described for β-catenin. The qRT-PCR primers used for Sox9 are as follows: forward; 5CAAGAACAAGCCACACGTCA 3′ and reverse; 5′CGCCTTGAAGATAGCATTAGGA 3′).

Supplementary Figure 2

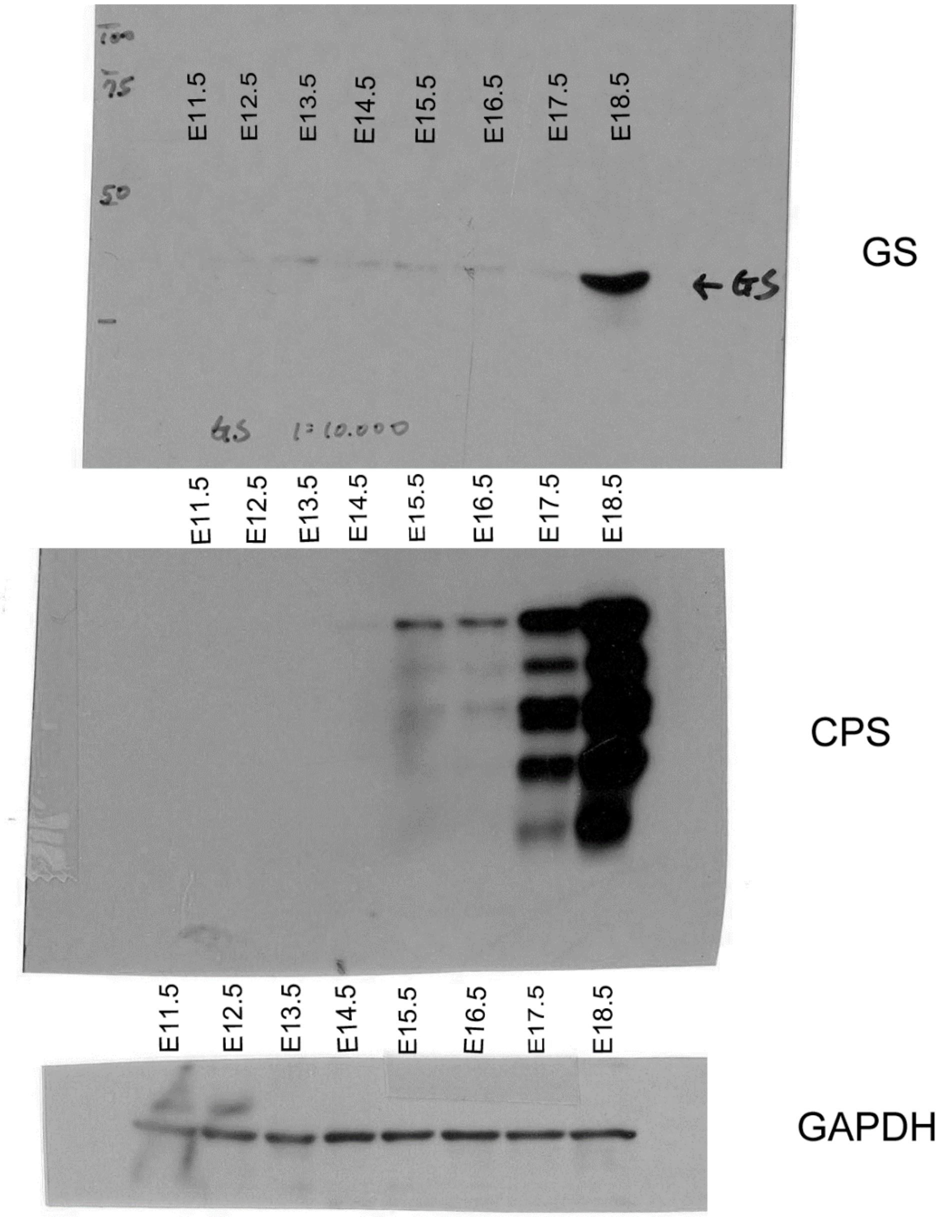

Supplementary Figure 2. Scanned images of original autoradiographs showing Western Blot analysis for GS, CPS and GAPDH in embryonic liver samples taken at the time points indicated. In the CPS blot the uppermost band corresponds to the 150kDa CPS protein, additional bands are believed to be degradation products. E11.5 samples were cropped out of the images shown in the main article (Figure 1A) as this time point was not included in the Immunohistochemical and Immunofluorescent analysis and their inclusion added little to the data we present.

Supplementary Figure 3

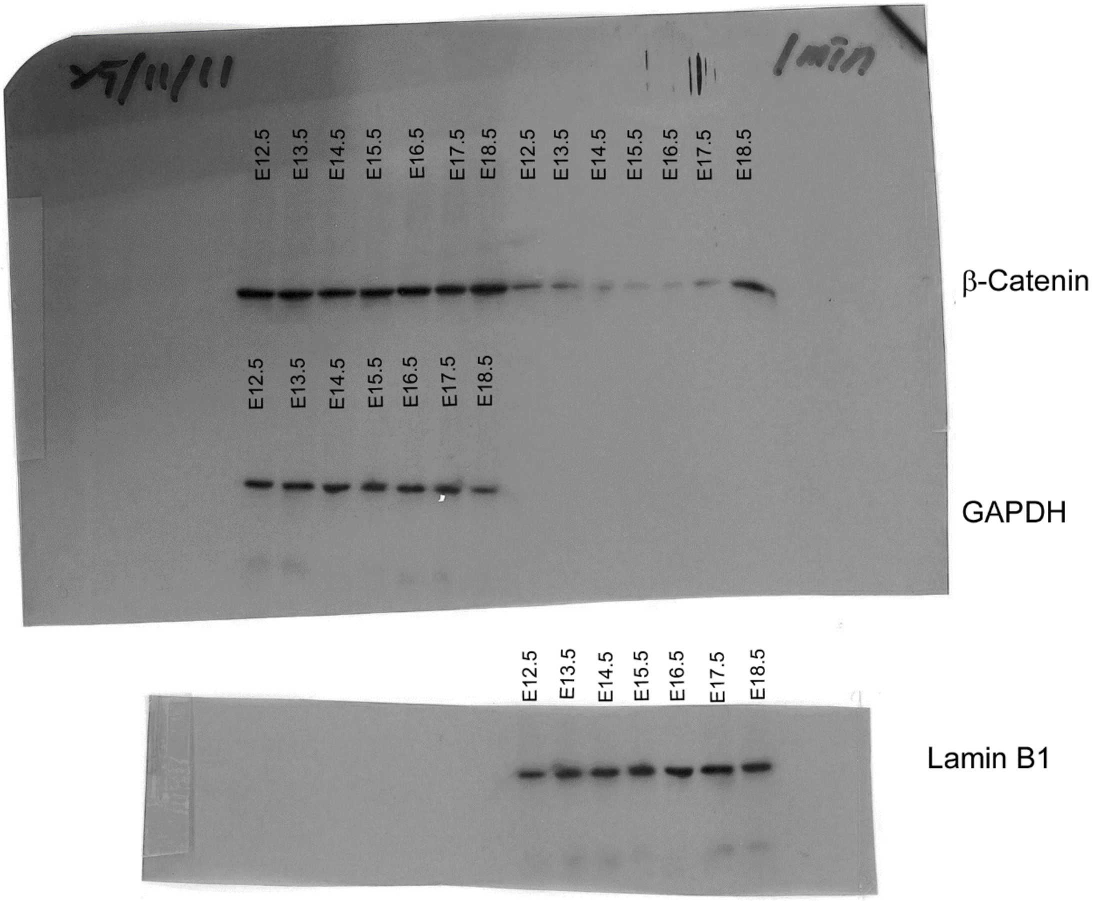

Supplementary Figure 3. Scanned images of original autoradiographs showing Western Blot analysis for β-Catenin, GAPDH and LaminB1 in embryonic liver samples taken at the time points indicated (see Figure 1B).
